# Associations Between Immigration-Related User Factors and eHealth Activities for Self-Care: Case of First-Generation Immigrants From Pakistan in the Oslo Area, Norway

**DOI:** 10.2196/11998

**Published:** 2019-08-16

**Authors:** Naoe Tatara, Hugo Lewi Hammer, Jelena Mirkovic, Marte Karoline Råberg Kjøllesdal, Hege Kristin Andreassen

**Affiliations:** 1 Department of Computer Science Faculty of Technology, Art and Design Oslo Metropolitan University Oslo Norway; 2 Center for Shared Decision Making and Collaborative Care Research Oslo University Hospital Oslo Norway; 3 Department of Community Medicine and Global Health, Institute of Health and Society Faculty of Medicine University of Oslo Oslo Norway; 4 Health Services Research Norwegian Institute of Public Health Oslo Norway; 5 Centre for Women's and Gender Research UiT The Arctic University of Norway Tromsø Norway; 6 Centre for Care Research Norwegian University of Science and Technology Gjøvik Norway

**Keywords:** immigrants, type 2 diabetes, self-care, information-seeking behavior, literacy, language

## Abstract

**Background:**

Immigrant populations are often disproportionally affected by chronic diseases, such as type 2 diabetes mellitus (T2DM). Use of information and communication technology (ICT) is one promising approach for better self-care of T2DM to mitigate the social health inequalities, if designed for a wider population. However, knowledge is scarce about immigrant populations’ diverse electronic health (eHealth) activities for self-care, especially in European countries.

**Objective:**

With a target group of first-generation immigrants from Pakistan in the Oslo area, Norway, we aimed to understand their diverse eHealth activities for T2DM self-care in relation to immigration-related user factors specific to this target group: proficiency in relevant languages (Urdu, Norwegian, English), length of residence in Norway, and diagnosis of T2DM compared with general user factors (age, gender, education and digital skills, and self-rated health status).

**Methods:**

Data were from a survey among the target population (N=176) conducted in 2015-2016. Using logistic regression, we analyzed associations between user factors and experiences of each of the following eHealth activities for T2DM self-care in the last 12 months: first, information seeking by (1) search engines and (2) Web portals or email subscriptions; second, communication and consultation (1) by closed conversation with a few acquaintances using ICT and (2) on social network services; and third, active decision making by using apps for (1) tracking health information and (2) self-assessment of health status. Using Poisson regression, we also assessed the relationship between user factors and variety of eHealth activities experienced. The Bonferroni correction was used to address the multiple testing problem.

**Results:**

Regression analyses yielded the following significantly positive associations: between Urdu literacy and (1) information seeking by Web portals or email subscriptions (odds ratio [OR] 2.155, 95% CI 1.388-3.344), (2) communication and consultation on social network services (OR 5.697, 95% CI 2.487-13.053), and (3) variety (estimate=0.350, 95% CI 0.148-0.552); between length of residence in Norway and (1) communication and consultation by closed conversation with a few acquaintances using ICT (OR 1.728, 95% CI 1.193-2.503), (2) communication and consultation on social network services (OR 2.098, 95% CI 1.265-3.480), and (3) variety (estimate=0.270, 95% CI 0.117-0.424); between Norwegian language proficiency and active decision making by using apps for self-assessment of health status (OR 2.285, 95% CI 1.294-4.036); between education and digital skills and active decision making by using apps for tracking health information (OR 3.930, 95% CI 1.627-9.492); and between being a female and communication and consultation by closed conversation with a few acquaintances using ICT (OR 2.883, 95% CI 1.335-6.227).

**Conclusions:**

This study implies immigration-related factors may confound associations between general user factors and eHealth activities. Further studies are needed to explore the influence of immigration-related user factors for eHealth activities in other immigrant groups and countries.

**International Registered Report:**

RR2-DOI 10.2196/resprot.5468

## Introduction

### Chronic Diseases and eHealth Use for Self-Care Among Immigrant Populations

Immigration surge made many European nations linguistically and culturally more diverse than ever, which poses a challenge to the health systems [1]. Immigrant populations are more likely to have challenges related to health care access due to language barriers and cultural differences [2-9]. Belonging to an ethnic minority as well as having low literacy in the official language of the host country are shown to be associated with poor health [10,11]. Furthermore, many immigrant groups have a lifelong exposure to low socioeconomic position [12], which leaves them at high risk of chronic diseases [13]. Different studies show that immigrant populations are often disproportionally affected by chronic diseases, such as type 2 diabetes mellitus (T2DM) [7,14-17]. The World Health Organization Regional Office for Europe recognized the importance to address these challenges, and advocated in its health policy framework “Health 2020” [18] that eHealth should be successfully implemented and utilized to reduce social health inequalities [19].

However, eHealth content is often text-based and designed for the nation’s majority population, thus enhancing social health inequalities rather than reducing them [11,20-24]. Designing eHealth services also aiming to include minority user populations requires sensitivity to their language and cultural preferences [25]. For immigrant populations, recent studies show less extended use of eHealth for specific purposes within self-care than among host populations [26-31]. However, knowledge is limited and mainly from the United States regarding the association between eHealth use and user factors that are especially relevant to immigrants, such as language proficiency [28,30,32,33] or the length of residence in the host country [34]. Both measures are frequently examined in relation to immigrants’ health behavior, health status, and access to and utilization of health care services [4,5,35-42]. Given that eHealth use can be considered a part of health behavior and use of available health care resources, the association between eHealth use for self-care and host language proficiency versus proficiency in their primary language or length of residence would be worth investigating.

In this paper, we discuss how this can be done in an explorative study identifying variations in the use of eHealth for self-care of T2DM among first-generation immigrants from Pakistan in the Oslo area, Norway. Self-care is defined by the World Health Organization as “keeping health, prevention of and dealing with illness” [43]. Therefore, we target not only those who are given diagnoses of T2DM but also those who are engaged with the prevention of T2DM.

### Pakistani Immigrants in Norway

At the beginning of 2018, first-generation immigrants comprised 14.1% of the whole population of Norway [44]. Pakistanis are one of the biggest immigrant groups from non-Western countries. The first Pakistani immigrants arrived in Norway in the 1970s. Steadily, new immigrants are coming from Pakistan for family reunification or establishment of a new family with Pakistani immigrants or their descendants in Norway [45]. The majority of this population concentrates in the Oslo area [46]. The completed level of education among the first-generation Pakistani immigrants in Norway varies widely [47].

The primary language of this population is Urdu. The Urdu language is written in the Urdu alphabet, which is unlike the Roman alphabet [48]. To our best knowledge, there is no statistic regarding Urdu literacy level of first-generation immigrants from Pakistan in Norway. However, data from United Nations Educational, Scientific and Cultural Organization shows the literacy rate in Pakistan among the population aged 15 years and older was 57% in 2014, and there was a considerable gap between males (69%) and females (44%) [49]. Thus, a nonnegligible number of first-generation Pakistani immigrants who migrated to Norway after school age, especially women, may also have a limited literacy level in the Urdu alphabet despite speaking fluently. In addition, our target population often use Roman Urdu—the Roman alphabet used to express Urdu pronunciation [50]. Roman Urdu is especially useful when typing text by a QWERTY keyboard on an information and communication technology (ICT) device.

Norwegian is the official and dominant language in Norway. According to a survey among first-generation immigrants with different countries of origin by Statistics Norway in 2016, 55% of the Pakistani participants self-reported high or very high Norwegian literacy level, whereas only 12% reported low or very low Norwegian literacy level [51]. However, the same survey shows that the immigrants who came at a later stage of life (after age of 17) are less likely to self-report a high or very high Norwegian literacy level (42%) than those who came to Norway at an earlier stage (age at immigration 0-6 years: 97%; 7-16 years: 89%) [51].

Data are not found regarding proficiency in other languages among the population. However, given that English is the most used language for eHealth content globally, those with English proficiency may use eHealth for T2DM self-care to a larger extent than those without English proficiency.

It has been demonstrated that the prevalence of diabetes is considerably higher among Norwegian-Pakistanis (women: 26.4%; men: 20.0%) than among their ethnic Norwegian counterparts (women: 2.7%; men: 6.4%) [14]. The reasons for the high diabetes risk are interrelated factors including both epigenetics and lifestyle; most of the diagnosis cases are T2DM [52]. Many experience significant changes in lifestyle when moving to Norway, including dietary changes [53]. Pakistani immigrants with diabetes have reported advice from Norwegian health care workers to be inadequate and difficult to comply with due to cultural differences [54].

### Objectives

In this exploratory study, we examine how the immigration-related user factors specific to this target group are associated with their diverse eHealth use for T2DM self-care compared with general user factors that are typically used in similar studies, such as age, gender, education, digital skills, or general health status.

Relevant immigration-related user factors to this target group include proficiency in the relevant languages (literacy in Urdu as their primary language; proficiency in Norwegian as the host language; having proficiency in other languages, such as English, because it is the most common language used for eHealth content globally), length of residence in Norway, and diagnosis of T2DM. In this paper, we refer to them as “target group-specific user factors.”

## Methods

### Description of Survey

This study is part of a larger survey carried out among first-generation Pakistani immigrants in the Oslo area (N=176) in 2015-2016 [50] to explore the use of eHealth in this population. The informants were recruited by purposive sampling with a multirecruitment strategy [55]. The inclusion criteria were (1) immigrated from Pakistan after the age of 18 years, (2) live in the Oslo area, (3) speak Urdu (the official language of Pakistan) as the primary language in their private life, (4) aged between 25 and 59 years, (5) have access to or interest in using ICT tools (PC, tablet, or smartphone) connected to the internet in daily life, and (6) motivated for and capable of activities for self-care of T2DM [50]. The first and third criteria reflect our intention to include those who are more likely than others to have language and cultural barriers to regular health services.

Ethical approval was given to the project protocol by Norwegian Social Science Data Services in June 2015 (project number: 43549). The details of the survey have been described elsewhere [55].

### Variables

#### Independent Variables

The independent variables can be divided into two categories: target group-specific variables and general variables. The target group-specific variables included literacy in Urdu, language proficiency in Norwegian, proficiency in another language (for each language), length of residence in Norway, and diagnosis of T2DM. The general variables included gender, age, self-assessment of health status, and completed education (both in Pakistan and Norway) in the form of total number of years. We also included frequency of asking for help when using ICT as a measure of digital skills as a part of the general variables.

We chose to use self-reported language proficiency by following the convention of similar studies of immigrants’ health [3,5,33,56-58]. Confidence in Urdu literacy and in the Norwegian language was assessed using a 5-point Likert scale. One of the survey’s inclusion criteria was speaking Urdu as the primary language in daily life [50]. However, as described in the Introduction, a nonnegligible proportion of participants may not have high-level literacy in Urdu despite speaking in Urdu daily. Also, Roman Urdu is often used for using ICT in general. Thus, for Urdu literacy, we were interested in reading and writing skills in both Urdu alphabet and Roman Urdu. For Norwegian language proficiency, we asked about confidence level in speaking as well as writing and reading. As there was no presumption about our target populations’ proficiency in other languages, the survey included a question asking informants to name any other languages that they had confidence in. Having proficiency in another specific language was based on each named language to this question (dichotomous variable).

The informants specified the length of their residence in Norway to one of the categories of 0 to 1 year, 1 to 5 years, 5 to 10 years, and more than 10 years. Using ranges of years rather than exact numbers follows the convention of similar studies [3-5,37,40,56].

Age was represented by a middle year of an age range because the survey used age range instead of actual age to avoid the risk that an informant could be identified by a combination of answers to survey questions.

Self-assessment of health status was obtained by a multiple-choice question ranging from poor=0 to excellent=5. The frequency of asking for help when using ICT was given by a multiple-choice question with alternatives ranging from never=0 to always=4. This question captured a lack of digital skills. Further details are described elsewhere [55].

#### Dependent Variables

We considered the same dependent variables that were used in our previous study [55]; dichotomous answers to nine questions asking usage of different types of eHealth for T2DM self-care in the last 12 months, and the total number of positive answers. Among the nine questions, three questions received few positive answers. The three questions were asking about having experience of the following: seeking relevant information with T2DM self-care by searching for software programs on PCs or mobile apps that could be used as a look-up tool (8/176, 4.5%), communicating or consulting about T2DM on portals for peer-communication (9/176, 5.1%), and communicating or consulting about T2DM self-care by online consulting to experts in diabetes (1/176, 0.6%). With such few positive answers, reliable statistical analysis was not possible and we decided to do further analyses only for the rest of the dependent variables. First, for seeking T2DM-relevant information (1) by using search engines that require input of search terms or (2) on specific websites or by email subscriptions that can be navigated by only scrolling and clicking. Second, for communicating or consulting about T2DM self-care (1) by using ICT in general for closed conversation with a few specific acquaintances, such as voice, video, or text communication, or (2) via social networking service (SNS). Third, for active decision making on T2DM self-care by using Web or mobile apps (1) for tracking health information, such as diet, physical activity, weight, blood glucose level, and so on, or (2) to assess own health status with regard to T2DM [55].

### Statistical Analyses

Logistic regressions were used to assess the relationship between each type of eHealth use with the independent variables described previously. For total eHealth usage, which was a count variable, we used Poisson regression.

To select the best regression models, all possible combinations of including and excluding independent variables were evaluated using the best-fit method in the statistical program R [59]. We used the Akaike information criterion (AIC) as the selection criterion between models [60]. The AIC awards models with a good fit to the data and at the same time punishes models that use many independent variables to avoid overfitting. Thus, the best model will have a trade-off between the fit to the data and the number of variables included in the model.

Our previous study revealed that the scores for the questions “total years of education in Pakistan and Norway” and “frequency for asking for help when using ICT” were strongly correlated [55]. Therefore, we defined the variable “education and digital skills” as the mean score of these two questions. Before computing the mean, the following two steps were taken. First, to take into account that they were on different scales, the scores of the variables were standardized. Further, the scores of the frequency of asking for help when using ICT were multiplied by −1 because higher knowledge results in less frequency of asking. The internal consistency of the scores for these two questions, using the standardized Cronbach alpha [61], was .70.

The questions about reading and writing in Roman Urdu and Urdu alphabet all reflect Urdu literacy. The internal consistency of the scores for these four questions, using the standardized Cronbach alpha, was .85. Therefore, we defined the “Urdu literacy” variable as the mean of these variables. Scores for the questions about reading, writing, and speaking the Norwegian language also showed a high internal consistency (Cronbach alpha=.92). Therefore, we also defined the “Norwegian language proficiency” variable as the mean of the three variables.

To reduce the number of variables to estimate in the statistical model and take into account that time is a continuous variable, the categorical variables for the length of residence (0-1 year, 1-5 years, 5-10 years, and more than 10 years) were transformed to a continuous variable by setting each of the variables to 0.5, 3, 7.5, and 15 years, respectively. If one immigrates at the age of 18 years or older, it is reasonable to expect that the effect of the length of residence tails off with time (eg, one learns more about the Norwegian society the first year after arrival compared to after the tenth year). Therefore, in addition to using the length of residence directly in the regression models, we also evaluated two transformations: the square root and the logarithm of the length of residence. By comparing the resulting models based on the AIC, the logarithm transform resulted in the best models and was used in the analyses.

A total of seven models were used to draw conclusions. To address the multiple testing problem, a Bonferroni-corrected significance value of 0.05/7=0.00714 was used in this paper.

## Results

### Characteristics of the Sample

The distribution of the informants by the data concerning the general variables and the dependent variables has been described elsewhere [55] and is reproduced in Table 1.

**Table 1:** Descriptive characteristics of the survey informants (N=176).^a^

Variables			Informants, n (%)
**Demographic variables**			
	**Gender**		
		Male	42 (23.9)
		Female	134 (76.1)
	**Age group by birth year range**		
		1981-1990	54 (30.7)
		1971-1980	61 (34.7)
		1956-1970	61 (34.7)
	**Total years of education from Pakistan and Norway**		
		0 years	14 (8.0)
		5 years	13 (7.4)
		<10 years	17 (9.7)
		<12 years	33 (18.8)
		<14 years	39 (22.2)
		≥14 years	55 (31.3)
	**Self-assessment of health status (score)**		
		Excellent (5)	11 (6.3)
		Very good (4)	27 (15.3)
		Good (3)	70 (39.8)
		Fair (2)	37 (21.0)
		Going up and down (1)	19 (10.8)
		Poor (0)	12 (6.8)
	**Frequency of asking for help when using ICT^b^**		
		Always (4)	18 (10.2)
		Often (3)	26 (14.8)
		Sometimes (2)	51 (29.0)
		Seldom (1)	12 (6.8)
		Never (0)	68 (38.6)
**Experience of eHealth use for T2DM^c^** **self-care in the last 12 months**			
	**For seeking relevant information**		
		By using search engines that require input of search terms	35 (19.9)
		On specific Web sites or by email subscriptions that can be navigated by only scrolling and clicking	63 (35.8)
	**For communication and consulting**		
		By using ICT in general for closed conversation with a few specific acquaintances	84 (47.7)
		By social networking service	58 (33.0)
	**For active decision making on self-care by using Web apps or apps for:**		
		Keeping track of health information	25 (14.2)
		Self-assessment of health status	38 (21.6)
	**Total number (variety) of eHealth types experienced**		
		≥8	0 (0.0)
		7	2 (1.1)
		6	5 (2.8)
		5	7 (4.0)
		4	9 (5.1)
		3	28 (15.9)
		2	38 (21.6)
		1	46 (26.1)
		0	41 (23.3)

^a^This is a reproduction of Table 4 in our previous study [56]. There are modifications of labeling of the experience of eHealth use for T2DM self-care in the last 12 months as well as the omission of data not relevant to this paper.

^b^ICT: information and communication technology.

^c^T2DM: type 2 diabetes mellitus.

Table 2 shows the distribution of the informants by the survey data related to language, length of residence, and diagnosis of T2DM. A vast majority of the informants showed confidence in reading (162/176, 92.0% for a total of “strongly agree” and “agree”) and writing (152/176, 86.3%) in Urdu alphabet. The proportions of informants with confidence in reading (123/176, 69.9%) and writing (116/176, 65.9%) in Roman Urdu were smaller than for Urdu alphabet. Not a negligible number of informants expressed lack of confidence in reading (7/176, 3.9% for a total of “strongly disagree” and “disagree”) and writing (13/176, 7.4%) the Urdu alphabet. The proportions of the sample lacking confidence in reading (26/176, 14.8%) and writing (33/176, 18.7%) in Roman Urdu were even higher. The Urdu literacy score was mean 4.26 (SD 1.26).

Regarding confidence level in the Norwegian language, the numbers concentrate around “neither” or “agree” in all three skills. The Norwegian language proficiency score was mean 3.35 (SD 1.08).

In total, 91 informants (51.7%) named English as another language they had confidence in. Arabic and Punjabi were named by two informants each, which is too small of a number to be applied in the statistical analyses. Therefore, we interpreted the answer “English” as “having English proficiency” in the further statistical analysis.

The majority of the sample (123/176, 69.9%) had lived in Norway for more than 10 years. The sample included only one informant (0.6%) who had lived in Norway for less than a year. A total of 27 informants (15.3%) answered that they had been diagnosed with T2DM.

### Association Between User Factors and eHealth Use

Table 3 shows the Pearson correlation coefficients between the different variables. The highest correlation coefficient was .78 between Urdu literacy and education and digital skills (*P*<.001).

**Table 2:** Descriptive characteristics of the survey informants regarding target group-specific user factors (N=176).

User factors			Informants, n (%)
**Urdu literacy “I am very confident in”:**			
	**Reading Urdu alphabet**		
		Strongly agree (5)	147 (83.5)
		Agree (4)	15 (8.5)
		Neither (3)	7 (4.0)
		Disagree (2)	2 (1.1)
		Strongly disagree (1)	5 (2.8)
	**Writing Urdu alphabet**		
		Strongly agree (5)	137 (77.8)
		Agree (4)	15 (8.5)
		Neither (3)	11 (6.3)
		Disagree (2)	3 (1.7)
		Strongly disagree (1)	10 (5.7)
	**Reading Roman Urdu**		
		Strongly agree (5)	95 (54.0)
		Agree (4)	28 (15.9)
		Neither (3)	27 (15.3)
		Disagree (2)	6 (3.4)
		Strongly disagree (1)	20 (11.4)
	**Writing Roman Urdu**		
		Strongly agree (5)	94 (53.4)
		Agree (4)	22 (12.5)
		Neither (3)	27 (15.3)
		Disagree (2)	9 (5.1)
		Strongly disagree (1)	24 (13.6)
**Norwegian language proficiency “I am very confident in”:**			
	**Reading Norwegian**		
		Strongly agree (5)	26 (14.8)
		Agree (4)	71 (40.3)
		Neither (3)	46 (26.1)
		Disagree (2)	24 (13.6)
		Strongly disagree (1)	9 (5.1)
	**Writing Norwegian**		
		Strongly agree (5)	22 (12.5)
		Agree (4)	53 (30.1)
		Neither (3)	53 (30.1)
		Disagree (2)	33 (18.8)
		Strongly disagree (1)	15 (8.5)
	**Speaking Norwegian**		
		Strongly agree (5)	28 (15.9)
		Agree (4)	49 (27.8)
		Neither (3)	73 (41.5)
		Disagree (2)	18 (10.2)
		Strongly disagree (1)	8 (4.5)
**Any other language an informant is confident in**			
	English		91 (51.7)
	Punjabi		2 (1.1)
	Arabic		2 (1.1)
**Length of residence in Norway**			
	0-1 year		1 (0.6)
	1-5 years		20 (11.4)
	5-10 years		32 (18.2)
	More than 10 years		123 (69.9)
Type 2 diabetes mellitus			27 (15.3)

**Table 3:** Correlation coefficients between the independent variables for the statistical analyses and *P* values.

Variable	1	2	3	4	5	6	7	8	9
1. Being a female	—^a^								
2. Age	−.04								
3. Self-assessment of health status	−.23^b^	−.32^c^							
4. Norwegian language proficiency	−.25^b^	−.06	.22^b^						
5. Type 2 diabetes	−.02	.32^c^	−.33^c^	−.16^d^					
6. English proficiency	−.25^b^	−.29^c^	.22^b^	.50^c^	−.19^d^				
7. Education and digital skills	−.31^c^	−.37^c^	.43^c^	.54^c^	−.26^c^	.55^c^			
8. Urdu literacy	−.22^b^	−.34^c^	.38^c^	.51^c^	−.28^c^	.53^c^	.78^c^		
9. Logarithm of years of residence in Norway	.09	.59^c^	−.31^c^	.16^d^	.20^b^	−.18^d^	−.29^c^	−.21^b^	—

^a^Not applicable.

^b^*P*<.01.

^c^*P*<.001.

^d^*P*<.05.

Table 4 shows the estimates of six multiple logistic regression models. The table shows results for the best regression model (based on best fit) for each dependent variable. To interpret the table, for example, “for seeking relevant information by using search engines that require the input of search terms” represents the dependent variable for the first regression analyses and the next three rows show the independent variables for this model. Note that estimates in the logistic regression analysis are the logarithm of the odds ratio, but we transformed them to odds ratios in Table 4 to make it easier to interpret. Table 5 shows the results of the Poisson regression analysis. Again, the best regression model is shown.

In the models in which language-relevant variables remained, Urdu literacy and Norwegian language proficiency were positively related to use of different types of eHealth for T2DM self-care. Urdu literacy was positively associated with the following: seeking relevant information to T2DM self-care on specific websites or by email subscriptions, communication and consulting about T2DM self-care by SNS, and with the total number of eHealth types experienced. Norwegian language proficiency was positively related to use of Web or mobile apps for self-assessment of health. Having English proficiency was neither positively nor negatively related to any dependent variable.

Regarding years of residence in Norway, the logarithm of this value was positively associated with communication and consulting about T2DM self-care by using ICT for a closed conversation with a few specific acquaintances, and by SNS, as well as with the total number of eHealth types experienced. Having been diagnosed with T2DM was not related to any dependent variable.

The composite variable education and digital skills appeared positively associated with the use of Web or mobile apps for keeping track of health information. Being a female was positively associated with using ICT for closed communication and consultation about T2DM self-care with a few specific acquaintances. Age had no significant association with any dependent variable. Self-assessment of health status did not remain in any model.

**Table 4:** Logistic regression analysis of the association between eHealth use and user factors.

Dependent and independent variables			Odds ratio (95% CI)	*P* value^a^
**For seeking relevant information**				
	**By using search engines that require the** **input** **of search terms**			
		Intercept	0.022 (0.002-0.209)	.001
		T2DM^b^	3.576 (1.301-9.832)	.01
		Urdu literacy	1.649 (1.026-2.651)	.04
	**On specific Web sites or by email subscriptions that can be navigated by only scrolling and clicking**			
		Intercept	0.019 (0.002-0.144)	<.001
		Urdu literacy	2.155 (1.388-3.344)	.001
		Logarithm of years of residence in Norway	1.371 (0.966-1.947)	.08
**For communication and consulting**				
	**By using information and communication technology in general for a closed conversation with a few specific acquaintances**			
		Intercept	0.396 (0.199-0.788)	.008
		Being a female	2.883 (1.335-6.227)	.007
		Logarithm of years of residence in Norway	1.728 (1.193-2.503)	.004
	**By social networking service**			
		Intercept	0.002 (0-0.207)	.008
		Age	0.951 (0.903-1.001)	.06
		Having English proficiency	0.379 (0.166-0.863)	.02
		Urdu literacy	5.697 (2.487-13.053)	<.001
		Logarithm of years of residence in Norway	2.098 (1.265-3.480)	.004
**For active decision making on T2DM self-care by using** **Web** **or mobile apps for**				
	**Keeping track of health information**			
		Intercept	4.253 (0.256-70.507)	.31
		Age	0.909 (0.844-0.979)	.01
		Education and digital skills	3.930 (1.627-9.492)	.002
		Logarithm of years of residence in Norway	1.753 (0.983-3.127)	.06
	**Self-assessment of health**			
		Intercept	0.263 (0.023-2.951)	.28
		Age	0.935 (0.887-0.985)	.01
		Norwegian language proficiency	2.285 (1.294-4.036)	.004
		Having English proficiency	0.418 (0.154-1.139)	.09
		Education and digital skills	2.414 (1.104-5.28)	.03

^a^A Bonferroni-corrected significance value of 0.00714 was used to interpret the *P* values.

^b^T2DM: type 2 diabetes mellitus.

**Table 5:** Poisson regression analysis of the association between the variety of experienced eHealth activities and user factors.

Total number (variety) of eHealth types experienced	Estimate (95% CI)	*P* value^a^
Intercept	−0.158 (−1.321, 1.005)	.79
Age	−0.019 (−0.035, −0.003)	.02
T2DM^b^	0.310 (−0.012, 0.633)	.06
Having English proficiency	−0.209 (−0.462, 0.044)	.11
Education and digital skills	0.181 (−0.037, 0.399)	.10
Urdu literacy	0.350 (0.148, 0.552)	.001
Logarithm of years of residence in Norway	0.270 (0.117, 0.424)	.001

^a^A Bonferroni-corrected significance value of 0.00714 was used to interpret the *P* values.

^b^T2DM: type 2 diabetes mellitus.

## Discussion

### Principal Findings and Implications for Future Studies

This study explored diverse eHealth use for T2DM self-care by first-generation immigrants from Pakistan in the Oslo area in relation to two types of user factors: (1) target group-specific user factors including proficiency in relevant languages, length of residence in the host country, and a diagnosis of T2DM, and (2) general user factors including education level, age, gender, self-assessment of health status, and digital skills. The results of multiple regression analyses showed that in all the final models at least one of the target group-specific variables remained. In addition, in most models target group-specific variables were most strongly associated with eHealth use. Therefore, in our survey sample, the inclusion of target group-specific user factors yielded better-fitting models predicting use of eHealth for different purposes compared with only including general user factors.

Informants with higher Urdu literacy were more likely than those with lower Urdu literacy to seek relevant information on Web portals or obtain such information by email subscriptions, to use SNS for communication and consulting about T2DM self-care, and to use a wider variety of eHealth services. For these types of eHealth use, Norwegian language proficiency did not remain in the final models. Use of mother tongue as the first choice for seeking information of interest is probably a natural behavior, as long as users expect or know that they can reach the information they need. SNS is often used for keeping online connection and communication with people one already knows. Therefore, it is also reasonable to speculate that informants communicate by SNS with their Pakistani family, friends, or alike who share an interest in T2DM self-care.

Norwegian language proficiency was positively associated with only use of Web or mobile apps for self-assessment of health as a part of T2DM self-care. It is reasonable to speculate that informants who are fluent in Norwegian have a better chance to know and use such services provided in the Norwegian language and that Norwegian eHealth services are of value for this subgroup of immigrants. The result leaves a question about why Urdu language literacy was not associated with the use of these eHealth services. Implications here could be either that such services are unavailable in Urdu language or that the informants were unaware of them despite their availability. Thus, it will be worth further examining if the informants have ever tried to search the internet for or been aware of such services offered in the Urdu language. Given the results that Norwegian language proficiency and Urdu literacy were associated with different types of eHealth activities, it would also be worth further investigating the difference in information or advice they receive from the different channels as well as the difference in user experiences. Another interesting question to pursue in future studies would be if the use of this type of eHealth service provided in the Norwegian language can positively affect the overall social integration in the Norwegian society for minority populations and vice versa, as well as health behavior.

Approximately half of the sample answered they are also confident in English. Nevertheless, English proficiency was not associated with any type of eHealth use for T2DM self-care. It is difficult to speculate the reason for irrelevance between having English proficiency and eHealth use for T2DM self-care among the sample. However, the implication here is that the target population may not use eHealth content in English for T2DM self-care as much as those in the Norwegian or Urdu language. As described in the Introduction, there are only a limited number of studies available that have investigated the relevance between immigrant populations’ eHealth use and language proficiency or similar [28,30,32,33]. All these studies are from the United States, where English is the primary language. Because of this, as well as differences in study design, our results cannot be simply compared with these studies. However, most of the studied cases found having English proficiency or similar user factors to be associated with eHealth use or confidence regarding eHealth use [28,30,32,33]. The results call for the need for more knowledge by studying other immigrant groups in European countries where English is not the primary language of either the immigrant groups or the host country.

In its logarithm form, years of residence in Norway was positively associated with closed communication with a few acquaintances and use of SNS, as well as the total number of eHealth types experienced in the last 12 months. An interpretation here could be that the longer immigrants live in Norway, the more people they connect with who also are at high risk of, or having, T2DM. Further, they may be more exposed to eHealth possibilities than those with shorter length of residence in Norway, although such effect may not be linear given the relatively short history of eHealth and needs to be seen by controlling the age of users. Other studies about immigrants’ use of eHealth in the United States showed that the effect of length of residence on eHealth use is not monotonically increasing; the group in the middle segment showed the highest eHealth engagement compared with those who lived in the host country the shortest or the longest [30,34]. These studies in the United States cannot be simply compared with our study because they include immigrants regardless of age at immigration, whereas all the informants in this study immigrated to Norway after the age of 18. Also, the types of eHealth activity they explore are not exactly the same as this study. Nevertheless, given the natural relevance between age and years of residence, future studies with larger sample sizes should further explore the relation between these factors by, for example, segmenting the sample and comparing the subgroups with different lengths of residence.

Despite remaining in the final model of using search engines to seek relevant information on T2DM self-care and the variety of eHealth activities experienced, having a diagnosis with T2DM was not significantly associated with any type of eHealth activity. One of the inclusion criteria of the survey was “being motivated for and capable of performing activities for self-care of T2DM”; thus, having a diagnosis of T2DM in itself may not be relevant enough to differentiate users and nonusers of eHealth for self-care of T2DM among the survey sample. Self-assessment of health did not remain in any model. Being in good condition could be a consequence of—and being in bad condition could be the motivation for—using eHealth for self-care. Thus, self-assessment of health at the time of participation in a survey may not be a good indicator of eHealth use for T2DM self-care.

### Implications for Development and Dissemination of Future eHealth Services to Reduce Social Health Inequalities

The results present valuable insight about relevant user factors of diverse eHealth activities for T2DM self-care among one of the little-studied, vulnerable user groups. The yielded knowledge can be used to guide further research on designing, developing, and disseminating eHealth tools for this vulnerable population to achieve the goal to reduce social health inequalities.

Despite an inclusion criterion of being “motivated for and capable of activities for self-care of T2DM,” not all types of eHealth activities were common enough among the sample. The findings of this study indicate that there is a certain divide among the target population regarding eHealth activities. First, those with high Norwegian language proficiency, and those with high education and digital skills, are more likely to use apps for self-assessment of health and to keep track of health information by using apps, respectively. Second, those with high Urdu literacy were more likely to go online for relevant information seeking and communication via SNS in connection with T2DM self-care than those with low Urdu literacy. In addition, nearly half of the sample had used ICT for closed conversation with specific acquaintances for consulting about T2DM, which is strongly associated with the longer period of residence in Norway. The implication here could be the importance of disseminating reliable health information or self-care apps through their social network. Relevant studies on eHealth use by immigrant populations also support use of the target populations networks [34] as well as stakeholders to promote available eHealth services [30,62].

On the other hand, translation into English seems not to be equally relevant, as having English proficiency was found to have no association with eHealth use for this population despite that more than half the sample showed confidence in English. Hence, to reduce social inequalities through eHealth services, these need to be offered both in Norwegian and minority languages that are especially relevant to high-risk groups of social health inequalities. The importance of provisioning easy-to-read, culturally appropriate health information in their own language is also outlined in relevant studies [28,30,32]. The quality and amount of information in eHealth content provided in the nondominant language should not be deteriorated because such a problem is reported in relevant studies [63,64]. Immigrants are heterogeneous in terms of linguistic and cultural background. Provision of eHealth services in many minority languages that are adapted to the cultural background would be highly demanding and costly. Machine translation seems not to have reached a level of sufficient accuracy, but it could be useful when used in combination with human translation [65,66]. Moreover, further development of machine translation technology has great potential to enable provision of eHealth services in minority languages at less cost and at a more trustable level than today.

### Limitations

Limitations regarding the survey sample and the survey methods we reported in our previous study [55] apply to this study as well. In addition, we should note that this study may suffer from nonprobability sampling, albeit the multirecruitment method we took was more appropriate for recruiting the target population than ordinary probability sampling [67].

For this particular study, we should note the following. Data about English proficiency was obtained in an indirect manner by asking about any language that the informants were confident in. This method may have caused underestimation of the number of informants with high confidence in English proficiency to some extent but should not have caused overestimation. Given the results of the regression analyses, even if the number of informants with high English proficiency increases to some degree, the results would not change drastically.

Our study relates to one specific population in one specific context. We invite future research to explore if these findings could be replicated in other contexts and populations and explore potential generalizability of the results.

### Conclusion

The contribution of this study is advancing knowledge on user factors associated with diverse eHealth activities among one of the immigrant populations in European countries, which is a vulnerable and little-known user group. Particularly, this study showed the importance of examining the target group-specific user factors for their associations with experience of diverse eHealth activities when the target group is a vulnerable immigrant population. This study also implied that to facilitate and enhance eHealth use by immigrant populations, eHealth services should be provided in minority languages rather than using English as a common language for foreigners. Future studies are needed for a further understanding of other target user groups vulnerable to poor health or low socioeconomic position due to the same or similar reasons as our sample.

## Abbreviations

AIC: Akaike information criterion
ICT: information and communication technology
OR: odds ratio
SNS: social network service
T2DM: type 2 diabetes mellitus
